# Overexpression-Induced α-Synuclein Brain Spreading

**DOI:** 10.1007/s13311-022-01332-6

**Published:** 2022-12-13

**Authors:** Rita Pinto-Costa, Eugenia Harbachova, Pietro La Vitola, Donato A. Di Monte

**Affiliations:** German Center for Neurodegenerative Diseases (DZNE), Venusberg-Campus 1, Building 99, Bonn, 53127 Germany

**Keywords:** Parkinson, Animal models, Oxidative stress, Neuronal activity, Vagus nerve, Gut-brain axis

## Abstract

Interneuronal transfer of pathological α-synuclein species is thought to play an important role in the progressive advancement of Lewy pathology and increasing severity of clinical manifestations in Parkinson’s and other diseases commonly referred to as synucleinopathies. Pathophysiological conditions and mechanisms triggering this trans-synaptic spreading bear therefore significant pathogenetic implications but have yet to be fully elucidated. In vivo experimental models support the conclusion that increased expression of intraneuronal α-synuclein can itself induce protein spreading throughout the brain as well as from the brain to peripheral tissues. For example, overexpression of α-synuclein targeted to the rodent dorsal medulla oblongata results in its transfer and accumulation into recipient axons innervating this brain region; through these axons, α-synuclein can then travel caudo-rostrally and reach other brain sites in the pons, midbrain, and forebrain. When protein overexpression is induced in the rodent midbrain, long-distance α-synuclein spreading can be followed over time; spreading-induced α-synuclein accumulation affects lower brain regions, including the dorsal motor nucleus of the vagus, proceeds through efferent axons of the vagus nerve, and is ultimately detected within vagal motor nerve endings in the gastric wall. As discussed in this review, animal models featuring α-synuclein overexpression not only support a relationship between α-synuclein burden and protein spreading but have also provided important clues on conditions/mechanisms capable of promoting interneuronal α-synuclein transfer. Intriguing findings include the relationship between neuronal activity and protein spreading and the role of oxidant stress in trans-synaptic α-synuclein mobility.

**Supplementary Information:**

The online version contains supplementary material available at 10.1007/s13311-022-01332-6.

## Introduction

Intraneuronal accumulation of α-synuclein aggregates called Lewy bodies, and Lewy neurites are pathognomonic of Parkinson’s disease (PD) and other human synucleinopathies. In 2003, Braak et al. [1] reported a stereotypical pattern of brain distribution and advancement of these α-synuclein inclusions in post-mortem brains of individuals with incidental (i.e., without a clinical PD history) or symptomatic PD. The burden of α-synuclein pathology, as described by Braak and colleagues, was classified into six stages of increasing severity, each featuring the involvement of discrete, susceptible brain regions. Earliest lesions at stage 1 affected the olfactory bulb, anterior olfactory nucleus, and dorsal motor nucleus of the vagus nerve (nucleus of the X^th^ cranial nerve, DMnX). At later stages, the pathological process proceeded mainly through an upward course that, starting from the medulla oblongata, involved specific areas in the pontine tegmentum, then advanced toward mesencephalic and prosencephalic nuclei and, finally, reached the neocortex. Shortly after its original description, this Braak staging of α-synuclein pathology became the basis for a novel hypothesis concerning the pathogenesis of PD. The progressive spreading of α-synuclein lesions was proposed to reflect a neuron-to-neuron exchange of pathogenetic forms of the protein that, once generated within specific brain regions, would advance via anatomically interconnected pathways of susceptible cell populations [2–4]. The site(s) of apparent initiation of this pathological process also became the focus of special attention. In particular, early accumulation of α-synuclein lesions in the DMnX could not only be responsible for the caudo-rostral pattern of pathological spreading underlying the Braak staging but may bear other important implications. DMnX cholinergic neurons possess long efferent axons that, through the vagus nerve, reach a variety of peripheral tissues/organs (e.g., the heart, lungs, and digestive tract) and make contact with neurons of the peripheral nervous system (PNS). Thus, the DMnX may represent a relay center and the vagus nerve may act as a conduit for the long-distant transfer of α-synuclein pathology from CNS to PNS neurons and vice versa [2, 5, 6]. Supporting this possibility, Lewy inclusions have been described in peripheral tissues, such as the gastrointestinal wall, and early clinical manifestations of PD include symptoms attributable to altered parasympathetic function, such as constipation [7–9]. The hypothesis of interneuronal spreading of PD pathology, after its original formulation, gained further support through direct and indirect evidence of cell-to-cell α-synuclein exchange. Significant support was provided, for example, by post-mortem observations in brain specimens of PD patients who died several years after receiving fetal ventral mesencephalic transplants. Lewy body-like inclusions were found within grafted nigral neurons and interpreted as potential evidence of a propagation of pathogenic α-synuclein species from host into grafted dopaminergic neurons [10, 11].

Given the pathogenetic and therapeutic implications associated with the development and progressive advancement of α-synuclein lesions in PD, it is not surprising that a variety of in vitro and in vivo experimental systems were developed to mimic cell-to-cell protein transfer and spreading of α-synuclein pathology within the brain and between the brain and peripheral tissues. A widely used in vivo approach has been a direct intraparenchymal injection of inoculates containing pathological α-synuclein forms. In some instances, animals were injected with tissue homogenates/lysates prepared from the brains of synucleinopathy patients or α-synuclein transgenic mice [12, 13]. More often, studies involved the administration of α-synuclein fibrils generated from recombinant protein. Inoculations were made at specific brain sites (e.g., striatum and/or substantia nigra) or, peripherally, in the intestinal (e.g., duodenal) wall or into other tissues, such as skeletal muscles [14–18]. In general, investigations using α-synuclein-containing tissue homogenates or pre-formed α-synuclein fibrils (PFFs) have revealed that these inoculates possess remarkable toxic properties. For example, stereotaxic injections of PFFs targeting the dorsal mouse striatum were found to induce formation of Lewy body-like inclusions, loss of nigral dopaminergic neurons, and behavioral abnormalities in the form of motor function impairment [14]. A number of other observations made in these models bear important pathogenetic considerations. In the brain, intraneuronal α-synuclein lesions, once formed in proximity to the intraparenchymal injection site, progressively spread to interconnected brain regions. This pathological spreading was contingent upon the presence of endogenous α-synuclein, since no α-synuclein-containing aggregates were observed when inoculations were made in α-synuclein *null* (*Snca*^−/−^) mice [13, 14]. Distinct histopathological and behavioral phenotypes were described as a result of injections of α-synuclein fibrils with different structural characteristics (so called α-synuclein “strains”), possibly mimicking the heterogeneous pathology that characterizes different human synucleinopathies [19–21]. Taken together, these observations strongly supported an intriguing mechanism of neuron-to-neuron transfer of α-synuclein lesions. Due to conformational changes, α-synuclein would gain prion-like properties: misfolded protein seeds could then act as templates that “corrupt” normal endogenous α-synuclein, propagate the aggregate-prone conformation(s), and ultimately spread the ensuing pathology [22, 23].

Animal models that involve intraparenchymal injections of insoluble α-synuclein (PFFs or α-synuclein-containing tissue homogenates) have been reviewed in greater detail elsewhere [e.g., 24, 25]. Here, we plan to focus on a different, possibly complementary in vivo strategy to induce interneuronal protein exchanges and to mimic α-synuclein spreading within the brain and between the brain and peripheral tissues; this strategy is based on the hypothesis that increased levels of neuronal α-synuclein may promote its spreading [26]. The relevance of this hypothesis is underscored by several lines of clinical, epidemiological, and experimental evidence supporting a pathogenetic role of enhanced α-synuclein expression in triggering or facilitating PD development. Direct evidence of a relationship between elevated α-synuclein and human parkinsonism was originally provided by genetic studies showing parkinsonian symptoms as well as PD-like pathology (including nigrostriatal neurodegeneration and accumulation of α-synuclein inclusions) in individuals carrying multiplication mutations of the α-synuclein gene (*SNCA*) and displaying enhanced α-synuclein brain expression at both the RNA and protein levels [27, 28]. Interestingly, a gene dosage effect was noted in these patients, since *SNCA* triplication caused earlier-onset disease and a more severe phenotype than allele duplication [29]. Further genetic, epidemiological, and experimental evidence supports the notion that, while a lifelong α-synuclein elevation is itself capable of triggering PD-like pathology and clinical parkinsonism, more transient and/or less pronounced protein increases also bear pathogenetic implications. For example, polymorphisms in the *SNCA* promoter region, such as changes in the length of the dinucleotide repeat sequence REP1, were shown to affect α-synuclein levels in the blood and brain and, through this mechanism, could modulate PD risk and influence the severity of PD clinical manifestations [30, 31]. Laboratory evidence also indicated that robust changes in α-synuclein expression could be consequences of neuronal injury. Exposure of experimental animals to toxic agents, such as the parkinsonism-inducing toxicant MPTP or the herbicide paraquat, induced neurodegenerative effects in the mouse substantia nigra pars compacta (SNpc) that were paralleled by a sustained neuronal α-synuclein upregulation [32, 33]. Similarly, an enhancement of α-synuclein immunoreactivity, especially in the neuropil, was reported in aged mice between 1 and 9 weeks after traumatic brain injury [34].

Based on these premises underscoring the occurrence and pathogenetic relevance of increased α-synuclein expression, animal models were developed to specifically address the question: is there a relationship between α-synuclein overload, interneuronal protein exchanges and brain spreading of α-synuclein? Studies addressing this question will be described first. Then, we will discuss important findings concerning pathophysiological α-synuclein properties and mechanisms of α-synuclein transfer that were obtained using models of overexpression-induced spreading.

## Evidence of Overexpression-Induced Spreading: The “Vagal” Model

**Fig. 1:**
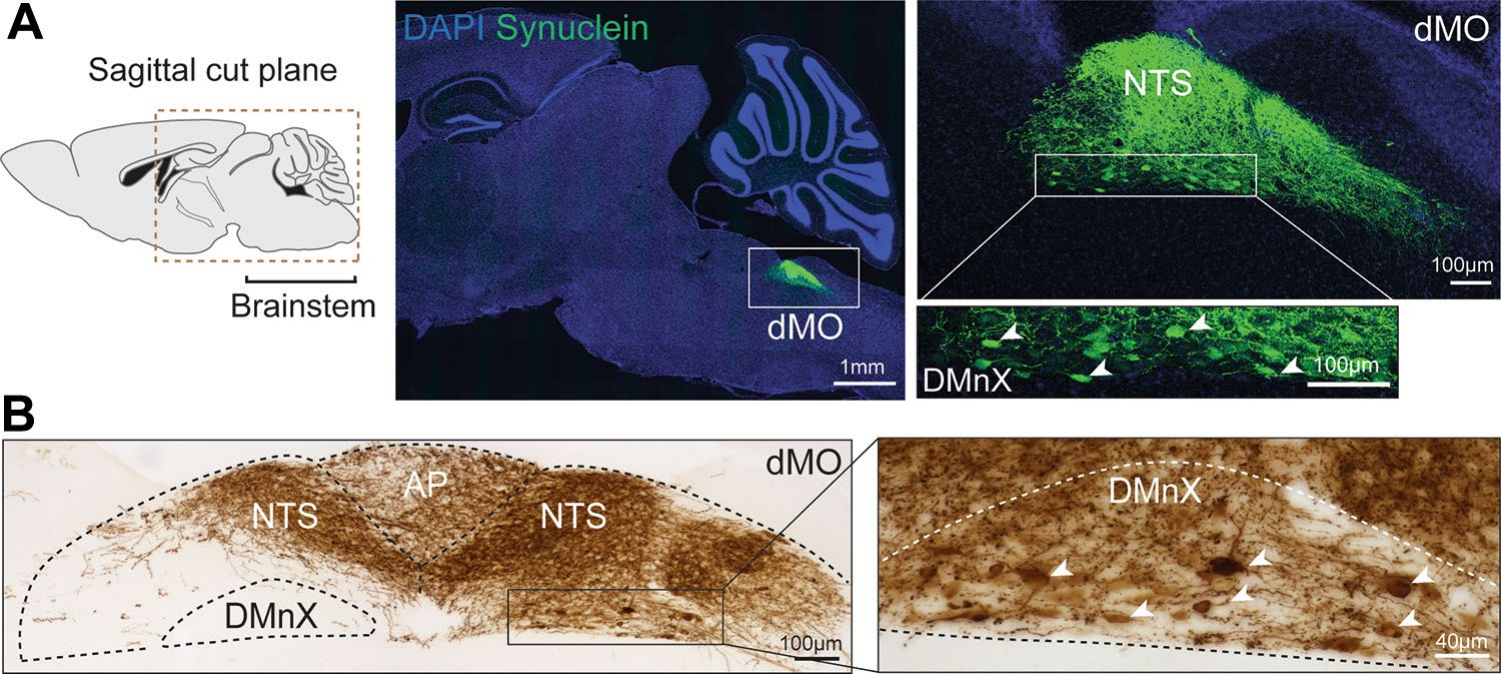
Targeted overexpression of hα-synuclein in the dorsal medulla oblongata. Images were obtained from mice that received an intravagal injection of hα-synuclein-AAVs and were sacrificed 2 weeks later. **A** The fluorescent image on the left shows hα-synuclein-containing neurons (green) in the dorsal medulla oblongata (dMO) in a sagittal brain section that was stained with both anti-hα-synuclein and DAPI (blue). The square rectangle delineates an area containing the nucleus of the tractus solitarius (NTS) and the dorsal motor nucleus of the vagus (DMnX) that is shown on the right top panel. The lower panel on the right shows transduced DMnX cell bodies at higher magnification (arrowheads). **B** Bright-field images show hα-synuclein-immunoreactive neurons (brown) in the DMnX, NTS and area postrema (AP) in a coronal section of the medulla oblongata. Arrowheads indicate transduced DMnX cell bodies in the high-magnification image

A reliable in vivo model for investigating α-synuclein transfer caused by enhanced protein expression should fulfill the basic criteria of inducing robust and consistent elevation of intraneuronal α-synuclein. Reliability and translational relevance of the model would also be improved, however, if this overexpression targeted neuronal populations and brain regions that are primary sites of α-synuclein pathology during PD development. For this reason, a “vagal” model was developed in which the potential caudo-rostral spreading of α-synuclein could be investigated after increased protein expression targeted to the dorsal medulla oblongata and, in particular, the DMnX (Fig. 1). A unilateral single injection of adeno-associated viral vectors (AAVs) into the vagus nerve of mice or rats was tested as a means to induce targeted overexpression [35, 36]. An incision made in the rodent neck allowed to expose the nerve and to inject AAVs delivering wild-type human α-synuclein DNA (hα-synuclein-AAVs). The rationale for transducing hα-synuclein included the consideration that a potential spreading of the transduced protein could be readily detected, thanks to the availability of antibodies that specifically react with the human form of the protein and would therefore allow to distinguish it from endogenous rodent α-synuclein. Intravagally injected AAVs induced a consistent pattern of robust hα-synuclein overexpression. This pattern followed the predicted anatomical distribution of axons forming the vagus nerve, namely, (i) efferent vagal axons that originate from neurons in the DMnX, and (ii) afferent axons stemming from perikarya in the vagal ganglia (in particular, the nodose ganglion) [37, 38]. Central projections of these latter ganglionic neurons terminate in the medulla oblongata where they innervate the solitary nucleus (or nucleus of the tractus solitarius, NTS). The injected AAVs were able to transduce both DMnX and ganglionic cell bodies, and the ensuing distribution of exogenous protein in the dorsal medulla oblongata (as revealed by immunohistochemical analyses using anti-hα-synuclein) was characterized by (i) protein accumulation within cell bodies and neurites of the DMnX ipsilateral to the side of vagal injection and (ii) marked immunoreactivity of neuronal fibers projecting into the NTS both ipsi- and (to a lesser extent) contralaterally (Fig. 1) [35, 36].

**Fig. 2:**
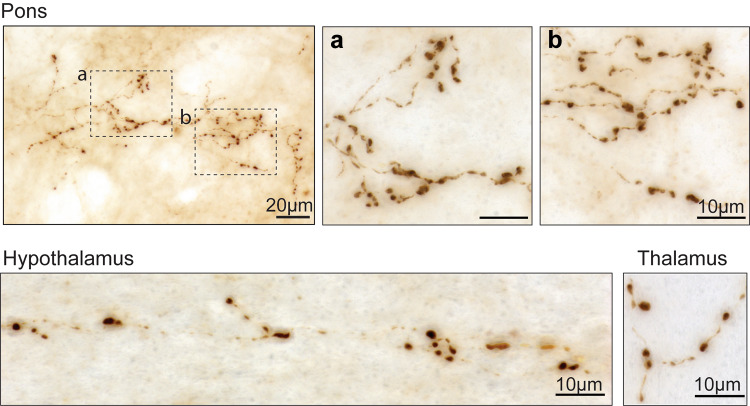
Neuronal accumulation of hα-synuclein as a result of caudo-rostral protein spreading. Images were obtained from rats that were injected intravagally with hα-synuclein-AAVs and sacrificed 6 weeks later. Coronal brain tissue sections were stained with anti-hα-synuclein. Bright-field images show immunoreactive axons (brown) with robustly labeled varicosities in the pons, hypothalamus, and thalamus

In the brain, axons forming the vagus nerve originate from or terminate in the medulla oblongata [37]. Therefore, following vagal AAV injections, overexpression of hα-synuclein should remain strictly confined within vagus-associated neurons in the lower brainstem. As a corollary to these anatomical considerations, detection of the exogenous protein in brain regions rostral to the medulla oblongata would denote its trans-synaptic jump and transfer into non-transduced recipient cells. Consistent with this latter scenario, a few weeks after intravagal administration of hα-synuclein-AAVs, hα-synuclein protein was first detected in tissue sections of the rat or mouse pons and subsequently found in specimens from the caudal and rostral midbrain and the forebrain [35, 36]. The exogenous protein was accumulated within dystrophic axons (Fig. 2). Quantitative analyses revealed that the number of these axons was highest in the pons and progressively lower in more rostral brain regions, indicating that areas closer to the medulla oblongata were more severely affected by the spreading pathology [35, 36]. Furthermore, when animals were injected with different titers of hα-synuclein-AAVs, a titer-dependent overexpression was observed in the medulla oblongata that resulted in a greater (higher titers) or less pronounced (lower titers) severity of caudo-rostral protein spreading [36]. These results underscore a direct relationship between levels of hα-synuclein within donor medullary neurons and extent of its transfer into recipient axons. Finally, a time-dependent effect was revealed by findings showing an increasing number of pontine, midbrain, and forebrain axons containing hα-synuclein at more advanced time points after the initial AAV transduction [35, 36].

Another critical observation concerning the spreading of hα-synuclein after its medullary overexpression was that it did not proceed randomly but rather followed a stereotypical pattern and sequence of topographical distribution [35, 36]. Preferred sites of protein accumulation included (a) the coeruleus–subcoeruleus complex in the pons; (b) the dorsal raphae, periacqueductal gray, and peripeduncular nucleus in the midbrain; and (c) in the forebrain, the hypothalamus in the diencephalon and the amygdala in the medial temporal lobe. Interestingly, all of these sites targeted by protein spreading are characterized by a common feature, namely, direct projections to the dorsal medulla oblongata [39, 40]. This finding is consistent with a process of hα-synuclein transfer involving anatomical interconnected pathways and occurring between medullary donor neurons and recipient axons innervating the dorsal medulla oblongata and connecting it to higher brain regions. Following intravagal hα-synuclein-AAV administration, protein spreading was observed throughout the brain hemisphere ipsilateral to the site of injection (e.g., left hemisphere after injections in the left vagus nerve); it also affected, albeit to a less pronounced degree, brain regions on the contralateral side [35]. A likely explanation for this bilateral distribution pattern is that the left or right dorsal medulla oblongata receives axons that originate not only from the ipsilateral but also (to a lesser extent) the contralateral brain hemisphere; through these axons, the spreading of hα-synuclein could affect both brain hemispheres [40]. Assessment of specific brain regions targeted by the spreading process included careful analyses in the SNpc, since nigral dopaminergic cells are highly vulnerable to both α-synuclein pathology and neurodegeneration in PD. No hα-synuclein immunoreactivity was detected, however, in the SNpc at time points up to 12 (in mice) or 18 (in rats) weeks post AAV administration [35, 36]. Although this observation may be perceived as a limitation of this model, several considerations could explain it. For example, neuron-to-neuron protein transfer in the “vagal” model may recapitulate pathophysiological events that, at least at an early stage, could spare nigral neurons. Subsequently, additional mechanisms may be needed for the spreading process, once initiated, to further expand and affect a greater number of neuronal populations, including nigral cells. Conflicting data have been reported concerning the presence of direct neuronal projections stemming from the SNpc and reaching the DMnX [41–44]. As mentioned above, all brain regions affected by hα-synuclein spreading in the vagal model feature direct anatomical connections to the dorsal medulla oblongata. Therefore, lack of strong SNpc-DMnX projections may preclude α-synuclein exchanges between donor neurons and recipient axons and consequently prevent spreading of the protein from the medulla oblongata to the SNpc.

The assumption that, in this vagal model, detection of hα-synuclein in brain regions rostral to the medulla oblongata was a consequence of interneuronal protein transfer needed to be validated by important control experiments and specific tissue analyses. In particular, these experiments and analyses were designed to rule out the possibility that, following the intravagal AAV injection, neuron-to-neuron passage of viral vectors would cause transduction and protein synthesis outside the medulla oblongata, thus accounting for or contributing to the observed extra-medullary accumulation of hα-synuclein protein. Several lines of evidence demonstrated lack of interneuronal AAV translocation and confinement of viral vectors within vagus-associated neurons in the medulla oblongata. For example, measurements of markers of AAV transduction, such as hα-synuclein or WPRE (an enhancer element incorporated into the AAV genome) mRNA, revealed robust expression in the dorsal medulla oblongata ipsilateral to the AAV injection, reflecting a unilateral transduction of DMnX cell bodies. As importantly, these markers were consistently absent in tissue specimens from the pons, midbrain, and forebrain [35, 36]. Further confirmation of targeted, confined transduction after intravagal AAV injections was obtained from experiments in which animals were treated intravagally with viral vectors that shared the same construct with hα-synuclein-AAVs (e.g., same promoter and enhancer elements) but carried DNA encoding a protein other than hα-synuclein. For example, analyses were performed in mice and rats injected with AAVs delivering green fluorescent protein DNA (GFP-AAVs) [35, 36]. The pattern of protein expression observed after GFP-AAV administration was similar to the distribution of hα-synuclein after treatment with hα-synuclein-AAVs; GFP protein was accumulated within cell bodies and neurites in the DMnX and within axonal projections innervating the NTS. The effects of GFP- *vs.* hα-synuclein-AAV injections were noticeably different, however, when evaluated in the pons, midbrain, and forebrain. Indeed, no GFP protein was ever detected in any extra-medullary location, underscoring the ability of AAV particles to transduce solely vagus-associated neurons and ruling out any potential interneuronal transfer of these particles [35, 36]. More recent control experiments were conducted using AAVs encoding for two synaptic proteins, namely, vesicle-associated membrane protein 2 (VAMP2) or synaptosomal-associated protein of 25 kDa (SNAP25). Results of these experiments were similar to the findings obtained with GFP-AAVs. Intravagal AAV injections caused VAMP2 or SNAP25 overexpression in the mouse dorsal medulla oblongata without triggering any caudo-rostral protein spreading (Di Monte et al., unpublished work).

Development and characterization of the α-synuclein vagal model, as described above, provided valuable insights into α-synuclein’s interneuronal mobility. It unequivocally demonstrated that increased α-synuclein expression is sufficient to trigger neuron-to-neuron transfer and retrograde axonal spreading of the protein. This spreading occurred via anatomically interconnected routes and, starting from the lower brainstem, followed a caudo-rostral pattern reminiscent of the upward advancement of α-synuclein pathology in PD. A comparison of α-synuclein’s behavior with the behavior of other proteins (GFP, VAMP2 or SNAP25) indicated that, following their AAV-induced transduction in the dorsal medulla oblongata, interneuronal mobility was not a mere consequence of increased protein expression; it rather represented a distinctive α-synuclein feature, likely due to properties of the protein yet to be fully elucidated. Following its initial characterization, the vagal model was used to investigate other important pathophysiological and mechanistical aspects of α-synuclein spreading. Results of these investigations will be described in the following paragraphs.

## Features and Mechanisms of α-Synuclein Spreading in the Vagal Model

### The Importance of Neuronal Integrity and Activity

One of the first questions raised by the observation of α-synuclein overexpression leading to its transfer from donor to recipient neurons related to the possibility that neuronal injury may induce or facilitate this process. It could be hypothesized that damage or degeneration of the overexpressing cells may promote the release of α-synuclein into the extracellular space where it would become available for internalization into nearby neurons. An initial answer to this question came from a study in which caudo-rostral hα-synuclein spreading was compared between two groups of rats that were injected intravagally with two separate hα-synuclein-AAV preparations [45]. Both preparations had the same viral construct and were manufactured using identical procedures, excepting for the methods used for their purification. Vector titers were adjusted to achieve similar transduction efficiency and similar levels of protein overexpression in the dorsal medulla oblongata. It was interesting, therefore, that the two treatments caused markedly different effects. Neurotoxicity and degeneration of DMnX neurons were only observed after injection with one of the two AAV preparations, most likely due to contamination with impurities (e.g., empty capsids) after a less effective purification process. Using the same “toxic” preparation, spreading of hα-synuclein was also significantly reduced, consistent with the conclusion that release from injured neurons is not essential for, nor does it increase, interneuronal protein transfer [45].

Further evidence of an inverse correlation between neuronal damage and α-synuclein brain spreading was provided by the results of a study investigating the long-term consequences of α-synuclein overexpression in the rat vagal model [46]. Sustained overexpression within donor neurons was itself deleterious. Starting at around 3 months after the initial intravagal AAV injection, the number of hα-synuclein-containing DMnX neurons progressively declined; this decrease was found to be due to neurodegeneration, with almost all of the transduced cells being lost at 1 year post-treatment. Caudo-rostral advancement of hα-synuclein was measured and followed over time in the same animals. Spreading progressed for several weeks after AAV administration and reached its peak at the 3-month time point. After then, no further advancement occurred, underscoring the notion that protein transfer was contingent upon continuous α-synuclein overexpression within relatively functional and active donor neurons [46].

Neuronal activity has been reported to modulate the pathogenicity and interneuronal mobility of α-synuclein as well as other proteins associated with human neurodegenerative diseases, such as amyloid β and tau [47–49]. Therefore, after establishing that cell integrity was essential for protein spreading under conditions of increased α-synuclein expression, another important question concerned the possibility that neuronal hyper- or hypoactivity may promote or, vice versa, suppress caudo-rostral α-synuclein advancement in the vagal model. This question was addressed in a study in which protein transduction within vagus-associated neurons was induced in conditional transgenic mice expressing hα-synuclein in a Cre recombinase-dependent manner [50]. When these animals were injected intravagally with AAVs carrying Cre recombinase DNA, targeted transgene expression was observed in the dorsal medulla oblongata. This expression reproduced the same anatomical distribution described in wild-type mice after intravagal administration of hα-synuclein-AAVs. The intravagal route was also used for injections of AAVs delivering DREADD (designer receptors exclusively activated by designer drugs) DNAs, in particular Gq-coupled hM3D DNA or Gi-coupled hM4D DNA [51, 52]. Targeted expression of either of these two DREADDS and systemic administration of the synthetic ligand clozapine-N-oxide (CNO) resulted in enhanced (hM3D) or suppressed (hM4D) activity of DMnX neurons [50].

To investigate the role of activity changes in the spreading process, mutant mice were injected with a solution containing Cre-AAVs (to induce hα-synuclein expression) together with either hM3D- or hM4D-AAVs (to express excitatory or inhibitory receptors). They were also treated with vehicle or CNO in order to assess and compare spreading of hα-synuclein under conditions of normal (vehicle-injected mice), enhanced or decreased neuronal activity. Cre-mediated expression of α-synuclein triggered its interneuronal transfer and caudo-rostral spreading in vehicle-treated animals. This effect was significantly modified by CNO administration. Increased or decreased protein spreading was indeed observed after CNO administration to hM3D- or hM4D-expressing mice, respectively, indicating a more or less pronounced α-synuclein transfer as a result of stimulation or inhibition of neuronal activity. In a final set of experiments, the effect of neuronal activity was also evaluated in relation to other pathological features of the vagal model, in particular the aggregation of α-synuclein triggered by its enhanced neuronal expression. Results of these experiments revealed that, besides enhancing protein transfer, neuronal hyperactivity significantly exacerbated intraneuronal aggregate pathology [50].

Evidence of a significant role of neuronal activity in modulating interneuronal α-synuclein transfer and protein aggregation bears a number of pathophysiological implications. Among others is the intriguing possibility that physiological traits that characterize specific neuronal populations and affect their activity may also render them more or less prone to α-synuclein exchanges and more or less involved in its pathological spreading. Neuronal populations, such as cholinergic DMnX neurons, dopaminergic cells in the SNpc and noradrenergic neurons in the locus coeruleus (LC), feature an autonomous pacemaking activity that is characterized by spontaneous rhythmic firing and is associated with high bioenergetic demands [53–55]. It is tempting to speculate that this activity-related physiological trait may facilitate α-synuclein exchanges involving these neurons; it could then contribute to their vulnerability to protein accumulation and aggregation and promote their role as important sites for the spreading of α-synuclein pathology.

### Pathological Consequences of Caudo-Rostral Spreading

Persistent transfer of hα-synuclein from medullary donor neurons into recipient axonal projections caused an early axonopathy that was characterized by whole axonal enlargement and the formation axonal swellings [35, 36, 46] (Fig. 2). These varicosities were irregularly spaced throughout the fiber treads, were intensely immunoreactive for hα-synuclein and contained aggregated forms of the protein that could be stained with the fluorescent dye Thioflavin S. Interestingly, the volume of spreading-induced axonal swellings augmented over time (e.g., between 8 and 18 weeks after vagal AAV administration) and was more pronounced in caudal (e.g., pontine) as compared to more rostral (e.g., midbrain) brain regions, indicating a progressive α-synuclein burden and more severe pathology in regions closer to the medulla oblongata [35, 46].

Evidence of axonal damage caused by neuron-to-neuron α-synuclein transfer raised the question of whether this injury may have long-lasting pathological consequences and, in particular, affect overall neuronal integrity. To address this question, detailed analyses were carried out in the LC [46]. This pontine nucleus contains noradrenergic neurons highly susceptible to α-synuclein pathology and neurodegeneration in PD [1, 56, 57]. The LC is known to send direct axonal projections into the dorsal medulla oblongata and, in the vagal model, is one of the pontine sites where hα-synuclein-immunoreactive fibers were consistently detected [35, 36, 39, 40]. Neuronal integrity was assessed in the rat LC at different time points after vagal AAV administration by stereological counting of Nissl-stained neurons. Neuronal counts were unchanged at 6 weeks post-treatment. A progressive cell loss was observed, however, at later time points, with the number of LC neurons being reduced by 3%, 11% and 15% at 3, 6, and 12 months, respectively [46]. Time-dependent neurodegeneration was accompanied by overt changes in the number and morphology of microglial cells and the number of astrocytes. When compared to age-matched control animals, a significant increase in microglial counts was found in the LC of AAV-injected rats at 3, 6, and 12 months, thus paralleling the time course of neuronal cell death. Tissue microgliosis was also characterized by an increased number of cells with morphological features of activated microglia; in particular, higher counts of IBA1-positive cells (IBA1 is a microglial marker) with a hypertrophic or amoeboid phenotype occupied the LC of AAV-treated animals. Staining of pontine sections with glial fibrillary acidic protein (GFAP) was used to assess astrogliosis and revealed a marked augmentation (approximately 100%) of immunoreactive cells at later time points (6 and 12 months) after vagal AAV administration [46].

Taken together, these findings clearly demonstrate that neuronal accumulation of α-synuclein as a result of protein transfer may bear pronounced pathological consequences. In the vagal model, the initial pathology predominantly affected axons, most likely reflecting their key role as recipients of the transferred protein. Long-term effects were also remarkable and included the degeneration of neurons in a brain nucleus (i.e., the LC) distant from the medulla oblongata but anatomically connected to it. In this nucleus, spreading-induced injury was accompanied by severe tissue reaction in the form of both micro- and astrogliosis. A final consideration stemming from findings in the vagal model relates to the apparent lack of α-synuclein accumulation within neuronal cell bodies that contrasted to the overt protein build-up within axonal projections. The precise reason(s) underlying these observations remain to be fully elucidated. It is conceivable that crowding and aggregation of α-synuclein within nerve fibers may induce axonal pathology while, at the same time, preventing efficient protein flow and access of α-synuclein into neuronal perikarya. Under these conditions, α-synuclein clearance may also become more effective within neuronal cell bodies than neuronal projections, further accounting for differences in protein accumulation between perikarya and axons. Finally, a dying-back process triggered by axonal damage may prompt cell demise prior to and in the absence of overt α-synuclein build-up within neuronal cell bodies.

### Mechanisms Promoting α-Synuclein Pathology in the Vagal Model: The Role of Oxidant Stress

Investigations into mechanisms that underlie interneuronal transfer and brain spreading of α-synuclein bear important implications not only from the standpoint of elucidating pathogenetic disease processes but also for the design of therapeutic strategies that may prevent and/or slowdown PD development and progression. A long-standing hypothesis concerning PD pathogenetic mechanisms implicates oxidant stress as a culprit or significant contributor to pathological processes; support for this hypothesis has recently been provided by findings in the vagal model. Initial experiments revealed that enhanced expression of hα-synuclein within DMnX neurons is itself associated with oxidant stress in the form of accumulation of reactive oxygen species (ROS) [58]. A relationship between intensity of oxidant stress and severity of α-synuclein pathology was then demonstrated by experiments in which mice first received a vagal injection of hα-synuclein-AAVs and were subsequently treated with the oxidant stress-inducing agent paraquat [58]. Enhanced ROS formation after this combined treatment caused a loss of DMnX neurons and significant α-synuclein alterations that included accumulation of nitrated and oxidatively modified forms of the protein and increased aggregation into oligomeric α-synuclein species. A key finding of this study was the demonstration of a direct link between oxidant stress and α-synuclein spreading. Indeed, assessment of hα-synuclein in tissue sections of the pons and midbrain showed counts of immunoreactive axons that were significantly higher in mice injected with hα-synuclein-AAVs and paraquat than in animals treated with hα-synuclein-AAVs and saline [58]. Microglial cells represented major sources of ROS production following AAVs/paraquat administration. This conclusion was supported by the results of experiments carried out in mice lacking functional microglial NADPH oxidase, a membrane-bound enzyme that catalyzes the one-electron reduction of oxygen to superoxide. When these mutant animals were injected with either AAVs/saline or AAVs/paraquat, the latter treatment failed to produce enhanced oxidant stress; at the same time, no increase in caudo-rostral spreading of hα-synuclein was observed in AAVs/paraquat-treated mice, indicating a direct relationship between microglia-induced oxidant stress and interneuronal protein transfer [58].

A separate recent study provided further evidence of oxidant stress as a mechanism promoting α-synuclein pathology in the vagal model [50]. As described above, this pathology is markedly affected by neuronal activity, with hyperactivity enhancing both hα-synuclein aggregation and spreading. Interestingly, evidence of increased ROS production was found when hα-synuclein-expressing neurons became hyperactive and, within these neurons, mitochondria were identified as likely ROS sources and key targets of oxidative damage. Rescuing experiments were then designed to test the hypothesis that, under these experimental conditions, mitochondrial superoxide dismutase 2 (SOD2) may play an important role in counteracting ROS accumulation and ensuing α-synuclein pathology. For these experiments, the vagal route of transduction was used to inject a cocktail of viral vectors that triggered co-expression of hα-synuclein, hM3D (an excitatory DREADD) and SOD2 within DMnX neurons. Results revealed that, in the presence of enhanced SOD2, hyperactivity-induced oxidative burden was significantly alleviated. As importantly, SOD2 transduction completely reversed the pathological effects of neuronal stimulation on α-synuclein aggregation and interneuronal transfer, providing compelling evidence of a mechanistic link between hyperactivity, oxidant stress and α-synuclein pathology [50].

In summary, results of studies using the vagal model strongly support a role of oxidant stress in PD pathogenetic processes. As importantly, they reveal a new toxic effect of enhanced ROS formation that was shown to promote interneuronal α-synuclein mobility. Exacerbation of α-synuclein spreading was observed after paraquat exposure and microglia-induced ROS production as well as during hyperactivity and mitochondria-related ROS burden. Data suggest therefore that oxidant stress is capable of increasing protein transfer regardless of the mechanism(s) causing this stress and irrespective of the site(s) of enhanced ROS formation. The new findings are also compatible with additive or synergistic effects that oxidant stress may have together with increased α-synuclein expression; together, they could exacerbate pathological processes relevant to PD development that include the formation of intraneuronal α-synuclein aggregates and the interneuronal spreading of pathogenetic α-synuclein species.

### α-Synuclein Species Involved in Protein Spreading

Different α-synuclein species may play a more or less pronounced role in spreading processes due to structural and biological properties that would confer them distinct interneuronal mobility. Shortly after the initial characterization of the vagal model, special attention was given to the possibility that misfolded protein species with prion-like properties may play an important role in mediating α-synuclein propagation from neuron to neuron and from brain region to brain region. A key experiment aimed at addressing this hypothesis was performed in α-synuclein null mice [36]. Lack of endogenous α-synuclein in these transgenic animals would be expected to prevent prion-like interactions between misfolded protein species and native α-synuclein; it should therefore abolish any spreading triggered by such interactions. Experimental data did not support this prediction, however. Intravagal injections of hα-synuclein-AAVs in α-synuclein null mice were found to trigger not only expression of the exogenous protein in vagus-associated neurons but also its spreading outside the medulla oblongata toward pontine, midbrain and forebrain regions [36]. These findings provided convincing evidence that, in the vagal model, interneuronal α-synuclein transfer does not occur through prion-like mechanisms and involves protein species devoid of seeding capability.

Another important series of experiments using the vagal model focused on the assessment of α-synuclein transfer/spreading in relation to protein aggregation and solubility [36]. In particular, the interneuronal mobility of monomeric, oligomeric, and fibrillar α-synuclein was compared in brain tissue sections from wild-type mice injected with hα-synuclein-AAVs. Sections were stained with conformation-specific antibodies capable of detecting total (monomeric and aggregated), aggregated (oligomeric and fibrillar), or only fibrillar (mature amyloid fibrils) α-synuclein. Results of these analyses, which were confirmed with additional staining procedures/assays (e.g., thioflavin S staining and proximity ligation assay), revealed that overexpressing donor neurons in the dorsal medulla oblongata contained all different forms (monomeric, oligomeric and fibrillar) of α-synuclein, whereas only monomeric and oligomeric α-synuclein were detected within recipient axonal projections [36]. Data therefore indicated that interneuronal protein transfer involved relatively soluble α-synuclein species, with amyloid fibrils having instead very limited (or absent) intercellular mobility.

Seeding-induced prion-like propagation and pathological accumulation of insoluble amyloid fibrils are features of the spreading process triggered by intraparenchymal injections of α-synuclein-containing tissue homogenates or PFFs (see “Introduction” section). These features contrast with findings in the vagal model that, as described in the previous two paragraphs, point to interneuronal transfer of soluble α-synuclein species devoid of templating capability. It is reasonable to conclude therefore that at least two mechanisms, prion- and non-prion-like, could contribute to the deleterious spreading of α-synuclein during PD pathogenetic processes. These two mechanisms are likely to play different roles under various pathophysiological conditions, but could also be interrelated and act in additive or synergistic fashion. An apparent limitation of the vagal model in mimicking PD-like α-synuclein pathology is the absence of *bona fide* Lewy inclusions in brain regions affected by overexpression-induced protein spreading. A possible scenario of interacting mechanisms could therefore be the following: during conditions of increased α-synuclein expression, initial transfer and accumulation of oligomeric α-synuclein may be followed by secondary or additional toxic “hits” that would induce formation of seeding-competent protein species and development of more pronounced aggregate pathology.

Evidence of a significant role of oxidant stress in promoting α-synuclein spreading in the vagal model provided intriguing clues on the interneuronal mobility of specific protein species. Regardless of whether oxidant stress was induced by exposure to paraquat or neuronal hyperactivity, enhanced ROS formation was consistently associated with accumulation of nitrated α-synuclein forms both within donor cells in the medulla oblongata and recipient axons in higher brain regions [50, 58]. Furthermore, when oxidant stress was attenuated by reducing neuronal activity or overexpressing the superoxide scavenging enzyme SOD2, intraneuronal levels of nitrated α-synuclein were also significantly decreased [50]. Taken together, these findings reveal a close relationship between oxidant stress, nitrated α-synuclein burden, and protein spreading. They also suggest that detection of nitrated α-synuclein is not only a marker of enhanced oxidative/nitrative reactions but may also distinguish neurons more actively involved in the spreading process. Another plausible interpretation of data showing a strict relationship between nitrated α-synuclein burden and protein spreading is that post-translationally nitrated α-synuclein species may themselves possess greater mobility and enhanced propensity to pass from neuron to neuron. This conclusion is supported by in vitro evidence showing that cell-to-cell α-synuclein exchanges were augmented after paraquat-induced oxidant stress and that this effect was completely prevented by addition of antibodies against nitrated α-synuclein to the incubation medium [58]. Intraneuronal accumulation of nitrated α-synuclein, which is primarily detected within Lewy inclusions, characterizes the brain of patients with PD or other human synucleinopathies, underscoring the translational relevance of investigations into the role of nitrated α-synuclein species in spreading processes [59, 60]. It is also noteworthy that evidence of a relationship between nitrated α-synuclein burden and enhanced protein transfer supports the development and evaluation of therapeutic strategies that, by targeting nitrated α-synuclein, could help counteract pathology propagation and disease progression in PD.

## Other Models of Overexpression-Induced Spreading

Besides the vagal model, other paradigms of overexpression-induced α-synuclein spreading have been developed and used to elucidate features and mechanisms of interneuronal protein transfer of likely relevance to PD pathogenetic processes. Spencer et al. [61] investigated pathological and behavioral consequences of a unilateral intraparenchymal injection of lentiviral hα-synuclein vectors into the hippocampus of non-transgenic, α-synuclein knock-out or transgenic (overexpressing human wild-type α-synuclein under the mThy1 promoter) mice. As a result of this treatment, they reported robust α-synuclein immunoreactivity within neuronal cell bodies and neuropil throughout the hippocampus not only ipsi- but also contralateral to the injection side. The latter finding was interpreted as a likely reflection of interneuronal α-synuclein transfer from pre-synaptic cells originating in the ipsilateral hippocampus to post-synaptic neurons in the contralateral hemisphere. Interestingly, lack of endogenous α-synuclein in knockout mice did not prevent this contralateral protein spreading, supporting the conclusion that, similar to results in the vagal model, trans-synaptic propagation did not require cross-seeding reactions. Pathological α-synuclein accumulation and trans-synaptic protein transfer were accompanied by axonal degeneration and behavioral deficits. All of these changes could be significantly counteracted by passive immunization with a monoclonal antibody recognizing amino acids 91–99 of the α-synuclein protein sequence [61].

Overexpression-induced α-synuclein propagation was also assessed as part of a study in which AAVs delivering human mutant A53T-α-synuclein were injected into the LC of wild-type mice [62]. This treatment caused α-synuclein phosphorylation, formation of proteinase K-resistant α-synuclein aggregates, and, ultimately, degeneration of noradrenergic LC neurons. When brain sections from these animals were stained with anti-hα-synuclein, robust labeling was detected within axons innervating output regions of LC neurons, such as the olfactory bulb, central amygdala, SNpc, and ventral tegmental area. Despite this widespread axonal protein distribution, no α-synuclein-positive cell bodies were observed outside the LC and, for this reason, trans-synaptic hα-synuclein propagation was ruled out as a feature of this model. It is noteworthy however that, as discussed above in relation to the vagal model, lack of α-synuclein accumulation within neuronal cell bodies may not necessarily rule out a trans-synaptic protein spreading that could only (or predominantly) become overt in the form of axonal α-synuclein burden. Another explanation for the apparent discrepancy between findings in the LC model *vs.* other models of overexpression-induced α-synuclein pathology concerns the possibility that a longer period of time and more sustained α-synuclein burden may be necessary to trigger overt spreading of α-synuclein after its overexpression in the LC [62]. Interneuronal α-synuclein mobility could be more or less rapid and pronounced depending upon a variety of conditions that include different neuronal populations, brain regions, and connecting pathways involved in the spreading process; these conditions are likely to vary and could generate distinct findings in different experimental models.

**Fig. 3:**
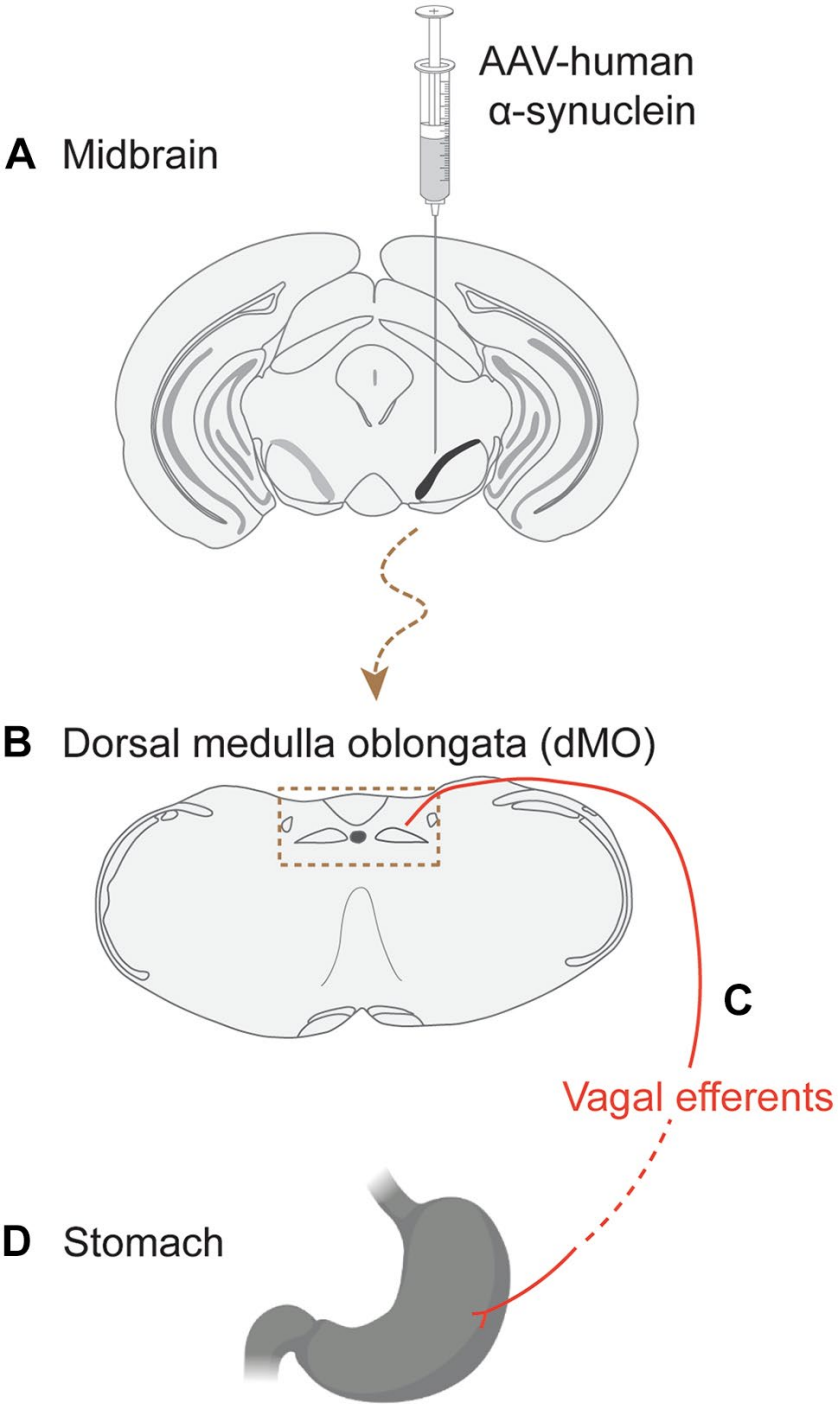
Schematic representation of long-distance brain-to-stomach hα-synuclein spreading. This spreading was initiated by an injection of hα-synuclein-AAVs in the rat midbrain (**A**). The exogenous protein first reached the dorsal medulla oblongata (dMO, box delineated by dashed lines) and, in particular, the dorsal motor nucleus of the vagus (**B**). It then traveled through efferent axons of the vagus nerve (**C**) and was finally detected within vagal nerve endings in the gastric wall (**D**)

Another clear example of a model of overexpression-induced α-synuclein spreading that provided valuable clues on α-synuclein’s pathological properties was generated to address the question of long-distance α-synuclein transfer between brain and peripheral tissues, in particular the gastrointestinal tract. As further discussed in the “Introduction” section, both clinical and experimental evidence of pathological α-synuclein lesions in the gut support the hypothesis that gut-to-brain and/or brain-to-gut pathways of α-synuclein spreading may play a role in PD pathogenetic processes [7, 8, 63, 64]. α-Synuclein propagation through a gut-to-brain pathway has been modeled experimentally through injections of PFFs or human PD brain lysate into the gastric and duodenal walls; this treatment resulted in a progressive accumulation of aggregated α-synuclein in a variety of brain regions, including the DMnX and substantia nigra [17, 65, 66]. On the other hand, evidence of brain-to-gut α-synuclein transmission was obtained using an in vivo model that involved an intraparenchymal injection of hα-synuclein-AAVs into the rat ventral mesencephalon immediately dorsal to the SNpc [6] (Fig. 3). Animals were sacrificed at 2, 6, and 12 months after this AAV injection and, throughout this period of time, tissue sections from the brain, vagus nerve, and stomach were analyzed for the presence of hα-synuclein. In the brain, the exogenous protein was accumulated in regions anatomically connected to the transduced midbrain areas (e.g., striatum, hypothalamus and LC). It was also detected in the medulla oblongata and, within the DMnX, an increasing number of hα-synuclein-loaded axons became visible between the 2-, 6- and 12-month time points. Quite importantly, some of these neuritic projections could be co-stained with an antibody against choline acetyltransferase, consistent with hα-synuclein transit through cholinergic vagal efferents. Immunoreactivity for hα-synuclein was absent in longitudinal sections of the vagus nerve stained with anti-hα-synuclein and analyzed at 2 months; labeled fibers could be detected, however, at 6 and 12 months, possibly reflecting a longer time interval needed for the protein to reach the vagus nerve from midbrain transduction sites. A similar time course of hα-synuclein staining characterized stomach whole mounts from AAV-injected rats. They were devoid of immunoreactivity at 2 months while showing evidence of hα-synuclein accumulation at later time points. The exogenous protein-labeled axons and terminal varicosities that encircled single neurons or small groups of neurons within ganglia of the myenteric plexus, revealing morphology and distribution typical of vagal motor nerve endings [6]. In summary, assessment of protein spreading in rats with midbrain hα-synuclein overexpression yielded a number of significant outcomes. It demonstrated long-distance propagation involving both intra- and inter-neuronal hα-synuclein transfer; the latter was clearly indicated by the detection of hα-synuclein within DMnX neurons and vagal efferent projections. It provided the first experimental evidence of α-synuclein’s ability to travel from brain to the gut; brain-to-stomach protein transfer occurred over a period of several months (up to one year), following a rostro-caudal pattern that progressively affected neurons in the brain, vagus nerve and gastric wall (Fig. 3). Finally, data revealed that the route of brain-to-stomach α-synuclein transmission preferentially (possibly, exclusively) involved vagal efferents, suggesting a unique propensity of these visceromotor fibers to accumulate α-synuclein and deliver it to long-distant peripheral sites.

A final paragraph of this discussion on animal models of overexpression-induced α-synuclein spreading will focus on transgenic mice. A large number of studies have investigated the effects of transgenic α-synuclein expression on α-synuclein pathology, albeit only in rare instances these studies were specifically designed to assess the relationship between protein overexpression and interneuronal α-synuclein transfer. No overt evidence of protein spreading has been reported by studies in α-synuclein transgenic mice to date [67]. These findings may indicate important differences in α-synuclein’s spreading potential in mice with “constitutive” (i.e., throughout development and postnatal life) α-synuclein overexpression as compared to animals in which protein overexpression is triggered “acutely” during adulthood. The interpretation of current knowledge about overexpression-induced α-synuclein spreading in transgenic mice warrants a few notes of caution, however. For example, it is important to note that a number of studies assessing α-synuclein pathology in transgenic mice were carried out in animals that expressed α-synuclein under the control of pan-neuronal promoters (e.g., PrP or Thy1) [68, 69]. In these mice, potential protein exchanges would be difficult to detect and might have been overlooked by earlier investigations due to the high levels of α-synuclein within both donor and recipient neurons. To avoid this confounding factor of robust and widespread transgene expression, alternative paradigms for assessing overexpression-induced spreading may be the use of transgenic mice with (i) α-synuclein expression driven by specific promoters (e.g., tyrosine hydroxylase promoters targeting catecholaminergic neurons), or (ii) inducible α-synuclein expression affecting distinct neuronal populations. Earlier publications of studies using transgenic mice with tyrosine hydroxylase-driven α-synuclein expression did not report any obvious presence of α-synuclein outside the neurons targeted for transgene expression [70, 71]. Other studies using animals with inducible α-synuclein expression did detect transgenic α-synuclein outside the targeted brain areas but attributed this finding to “leaky” expression of the transgene cassette [72, 73]. Overall, conclusive evidence in favor or against overexpression-induced α-synuclein spreading in transgenic mice is still lacking. Further investigations specifically designed to address this important experimental question are therefore warranted.

## Summary and Final Remarks

Changes in intraneuronal α-synuclein expression, transient or more permanent, are known to occur under a variety of pathophysiological conditions, underscoring the relevance of models and studies aimed at elucidating the consequences that these changes may have on disease pathogenetic processes. An important general consideration arising from work using models of overexpression-induced α-synuclein spreading is that any condition associated with α-synuclein overload represents a potential risk for interneuronal protein transfer and pathological α-synuclein propagation. Over the past decade, experiments using these models have yielded a number of other important findings that were described and discussed in previous sections of this review. For example, new data supported the concept that both prion- and non-prion-like mechanisms could contribute to the propagation of pathological α-synuclein species. This concept bears clear pathogenetic implications. The ability of α-synuclein to spread via multiple mechanisms underscores a higher risk that toxic α-synuclein species, once generated within a few neurons, may affect nearby cells and ultimately damage neurons throughout the brain. Different mechanisms of α-synuclein spreading, prion- and non-prion-like, could play different pathogenetic role under different pathophysiological conditions. It is also possible, however, that they may act additively or synergistically during different phases of development of α-synuclein pathology, raising scenarios of interacting toxic and pathological events that warrant greater attention by future investigations.

Other examples of important new findings described in this review include data revealing a relationship between protein expression, oxidant stress, and interneuronal α-synuclein transfer; accumulation of nitrated α-synuclein was identified as an important marker of this relationship. Moreover, findings in animals in which protein overexpression was initially induced in the midbrain provided clear evidence of brain-to-gut α-synuclein spreading that proceeded through vagal efferent fibers. Finally, experimental data supported the suitability and value of overexpression-induced spreading models for testing new therapeutics targeting α-synuclein pathology. Based on these premises, further work aimed at characterizing current models and developing new paradigms of overexpression-induced α-synuclein propagation is warranted. Future investigations using these models will likely contribute to unravel pathophysiological α-synuclein traits and to address key questions concerning pathogenetic processes, such as (i) the interrelationship between prion and non-prion α-synuclein pathology, (ii) the different propensity of various neuronal populations to protein accumulation and spreading, (iii) the distinct interneuronal mobility that may characterize different α-synuclein species, (iv) the role of α-synuclein spreading in neuronal damage/demise, and (v) mechanisms of α-synuclein transmission through the vagal route.

## Supplementary Information

Below is the link to the electronic supplementary material.

Supplementary file1 (PDF 499 kb)

Supplementary file2 (PDF 516 kb)

Supplementary file3 (PDF 524 kb)

Supplementary file4 (PDF 507 kb)

## Data Availability

Data used to generate the figures of this manuscript are available upon request.
